# Trends in Pediatric Cancer Care in Florida From 1981-2020: Changing Patterns in a Growing and Increasingly Diverse Population

**DOI:** 10.7759/cureus.35061

**Published:** 2023-02-16

**Authors:** Peter H Shaw, Jonathan Metts, Ernest Amankwah, Don E Eslin, Scott Bradfield, William B Slayton, Brian Hays, Brian Calkins, Juan Rico, Jeffrey Krischer

**Affiliations:** 1 Pediatrics/Oncology, Children's Wisconsin/Medical College of Wisconsin, Milwaukee, USA; 2 Pediatric Oncology, Johns Hopkins All Children's Hospital, St. Petersburg, USA; 3 Oncology, Johns Hopkins All Children's Hospital, St. Petersburg, USA; 4 Pediatric Oncology, St. Joseph’s Children’s Hospital, Tampa, USA; 5 Pediatric Oncology, Nemours Children's Health System, Jacksonville, USA; 6 Pediatric Oncology, University of Florida College of Medicine, Gainesville, USA; 7 Epidemiology, University of South Florida Morsani College of Medicine, Tampa, USA; 8 Pediatric Hematology Oncology, University of South Florida Morsani College of Medicine, Tampa, USA; 9 Epidemiology and Biostatistics, University of South Florida Morsani College of Medicine, Tampa, USA

**Keywords:** healthcare disparity, cancer epidemiology, clinical trial enrollment, cancer incidence, pediatric cancer

## Abstract

Background

The Florida Association of Pediatric Tumor Programs (FAPTP) has used the Statewide Patient Information Reporting System (SPIRS) since 1981 to track all new cases of pediatric cancer. We reviewed the last 40 years of data to see how pediatric cancer care has evolved.

Methods

We retrospectively analyzed SPIRS data from 1981 through 2020 in five-year increments, looking at numbers of new diagnoses, care delivery sites, and trial enrollment in Children’s Oncology Group (COG) studies.

Results

From 1981-2020 Florida’s population increased almost 88% while the pediatric population only grew 61%. New pediatric cancer diagnoses increased 326% to over 1,000 new cases/year. The percentage of patients treated at FAPTP centers grew from 30% to 57% with an annual percentage change (APC) of 10.3% (95% Confidence Interval [CI] of 0.6 to 20.9%). The rate of COG clinical trial enrollment decreased from 32% in 1981-1985 to 20% in 2016-2020, for an APC of 8.91% (95% CI of -13.3 to -4.3%).

Conclusions

The striking increase in pediatric cancer cases in Florida over the last 40 years was out of proportion to the population growth. More patients received care at FAPTP centers, but a lower percentage were enrolled on COG trials.

## Introduction

Overall, pediatric cancer cure rates have improved in the United States in the last 40 years, mostly due to incremental improvements forged by successive clinical trials run by pediatric cooperative groups. These groups included the Children’s Cancer Group (CCG), the Pediatric Oncology Group (POG), The National Wilms Tumors Study Group (NWTS) and the Intergroup Rhabdomyosarcoma Study Group (IRSG), all of which merged in 2000 to form the largest current pediatric cooperative oncology organization in the world, the Children’s Oncology Group (COG). In the mid-1970s, 58% of children aged 0 to 14 years and 68% of those adolescents aged 15 to 19 years diagnosed with cancer survived at least five years [1]. From the years 2010-2016, 84% of children and 85% of adolescents diagnosed with cancer were alive at five years from diagnosis, showing ongoing improvement [2]. Analysis of these data shows that the cancer mortality rate (the number of deaths due to cancer/100,000 people per year) among children and adolescents decreased by over 50% from 1975 to 2017 [2].

The National Cancer Institute’s Surveillance, Epidemiology and End Results (SEER) Program began collecting data in 1973 to better study cancer incidence and outcomes in the United States. It initially included Connecticut, Iowa, New Mexico, Utah, Hawaii and the Detroit and San Francisco-Oakland metropolitan areas. It has subsequently expanded to several other states and cities. These registries are not exclusively pediatric, but rather contain cancer patients of all ages.

Also in 1973, a statewide cancer initiative was started in Florida to focus on the pediatric population. The non-profit Florida Association of Pediatric Tumor Programs (FAPTP) was created to bring together pediatric cancer centers in the state to promote clinical and research collaboration. Since 1981, the FAPTP has used the Statewide Patient Information Reporting System (SPIRS) to track all new cases of pediatric cancer in the state from birth through 21 years of age. In the early 1990s, analysis of the data led to several publications looking at pediatric cancer incidence [3], patterns of care [4], and progress in care [5] in the state.

From 1981-2020 Florida’s population grew almost 88%. During that time FAPTP centers have increased in number from 13 to 16. As the SPIRS data have not been analyzed for almost 20 years, we re-examined the data to determine how pediatric cancer care has evolved on a state-wide scale in the last 40 years since the SPIRS was created. Clinical trial enrollment is part of the SPIRS data set, with patients being enrolled on studies through the CCG or POG through the year 2000, after which they merged into the COG.

This research was previously presented as a poster at the American Society of Pediatric Hematology-Oncology Annual Meeting, April 2021.

## Materials and methods

SPIRS includes registration and follow-up data collected for each patient diagnosed and treated at a FAPTP Center. Patients are registered in SPIRS from each of the 16 pediatric oncology centers and include all new children and young adults less than 21 years of age who present with malignancies. The patient data is submitted by data managers from each treatment site to the FAPTP Central Office in Tampa. Registration data includes demographic and diagnostic information about the new patient, while follow-up data represents a yearly check on the patient’s progress. We retrospectively analyzed the SPIRS data from 1981 through 2020, looking at the numbers of new diagnoses, care delivery sites, and trial enrollment in cooperative group studies (CCG/POG/COG). The new diagnoses included Florida residents as well as pediatric cases referred from other states or countries for treatment in Florida and that were treated at FAPTP centers. There was no distinction between biologic and therapeutic studies in the SPIRS database. The clinical trial enrollment data in the SPIRS only accounts for those patients who participated in the studies of these large groups. We examined the data in five-year increments starting in 1981-1985 and ending in 2016-2020. Joinpoint regressions were used to estimate annual percentage changes (APCs) with the corresponding 95% confidence intervals in log-transformed percentages. We were unable to perform the analyses by cancer type since the data used for analyses were aggregate data for all patients combined.

## Results

**Figure 1:**
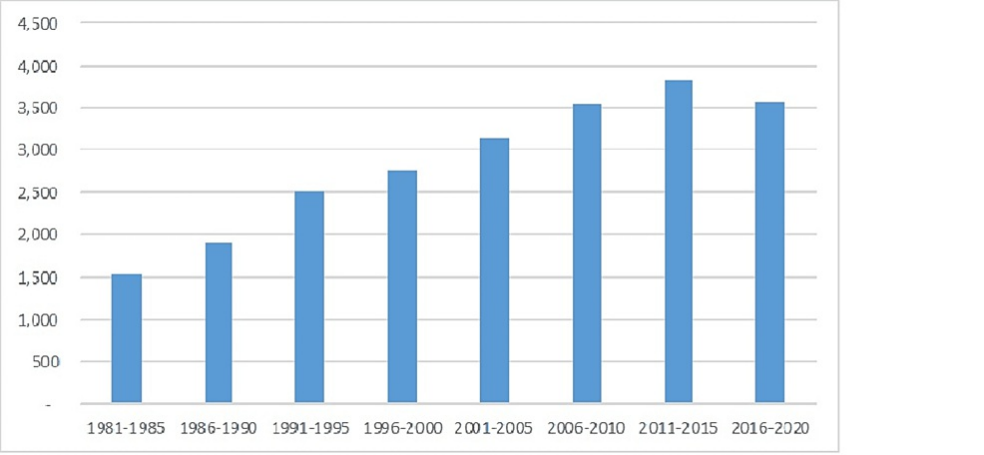
New Pediatric Cancer Cases in Florida

**Figure 2:**
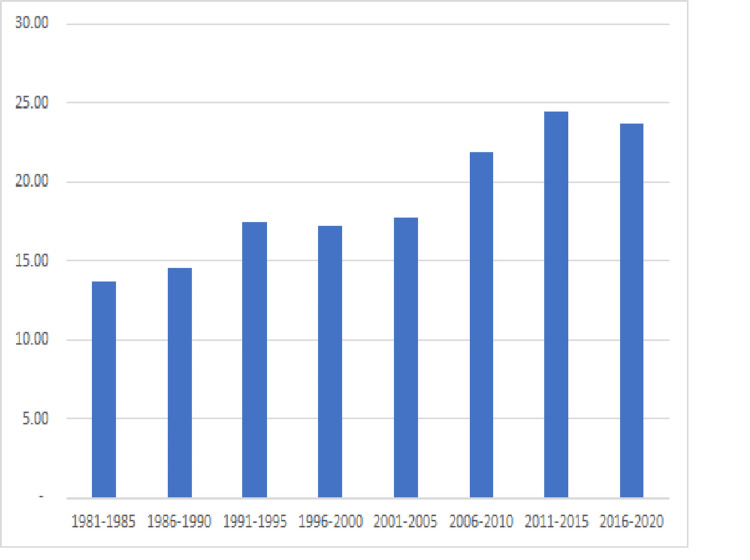
Pediatric Cancer Incidence in Florida/100,000 population

From 1981-2020 Florida’s population grew almost 88%, from 11.3 million to 21.3 million inhabitants. Simultaneously the population under 21 grew only 61% while new documented pediatric cancer diagnoses increased 326% to over 1,000 new cases/year over the years 2016 to 2020 (see Figure 1). This equates to an increase from 13.63 new cases per 100,000 population in 1981-1985 to 23.71 new cases per 100,000 population in 2016-2020 (see Figure 2). The median age of pediatric cancer patients increased over that time from 6 to 9 years old with a consistent gender breakdown of 55% male and 45% female patients.

**Figure 3:**
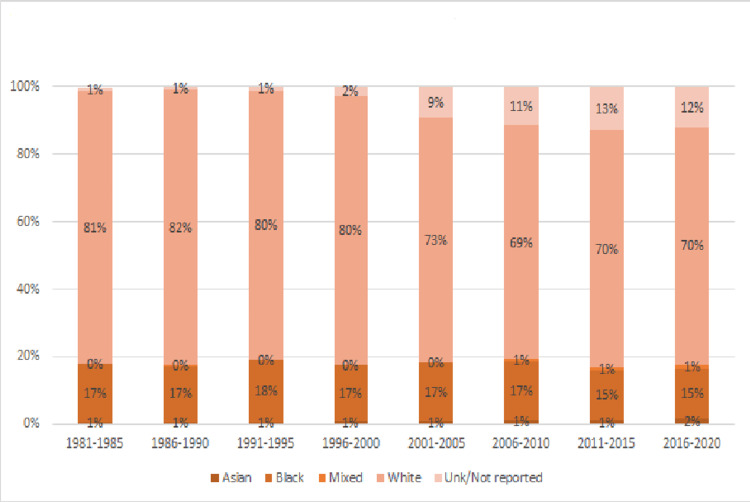
Racial Makeup of Florida Pediatric Cancer Patients 1981-2020

**Figure 4:**
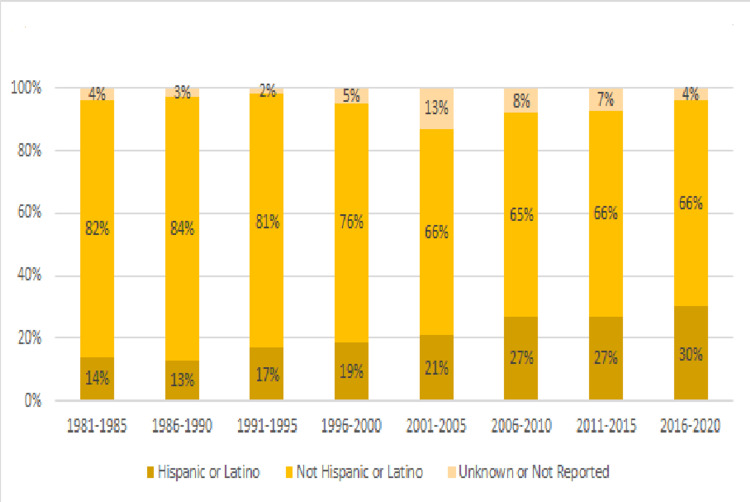
Ethnic Makeup of Florida Pediatric Cancer Patients 1981-2020

During this time, SPIRS data demonstrate that the population of patients became more racially and ethnically diverse. Between 1981 and 1985, 81% of the patients in the database were white, 17% black and 1% unknown or not reported, while between 2016 and 2020, 70% were white, 15% were black and 12% were unknown or not reported (see Figure 3). When looking at the ethnic breakdown as defined in SPIRS, from 1981 to 2020, we saw the proportion of Hispanic or Latino patients rise from 14% to 30% while those that did not identify as Hispanic or Latino decrease from 82% to 66% (see Figure 4). During this time, the state’s Hispanic population grew from 9% in 1980 to 26% in 2020. Asian patients made up 1% of the cancer patients and this increased to 2% in the most recent data set while those identified as “mixed race” rose from less than 1% to 1% of the patients.

**Figure 5:**
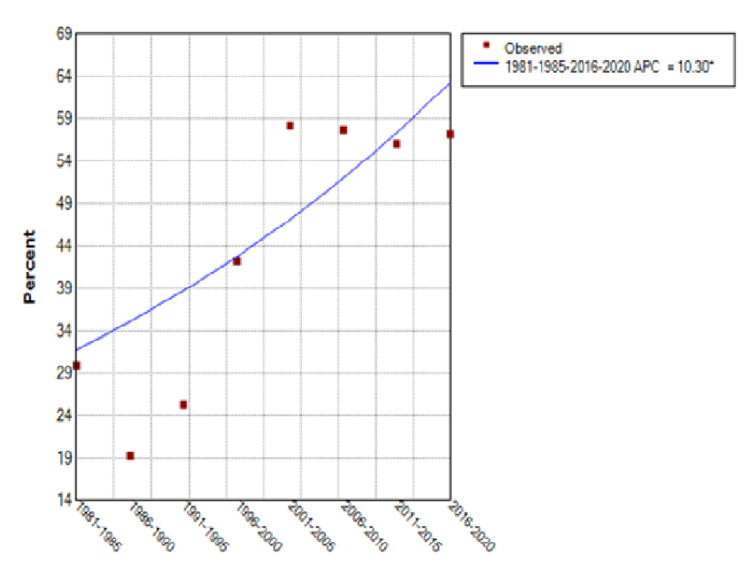
Patients Treated at Any FAPTP Center FAPTP: Florida Association of Pediatric Tumor Programs; APC: Annual percentage change

**Figure 6:**
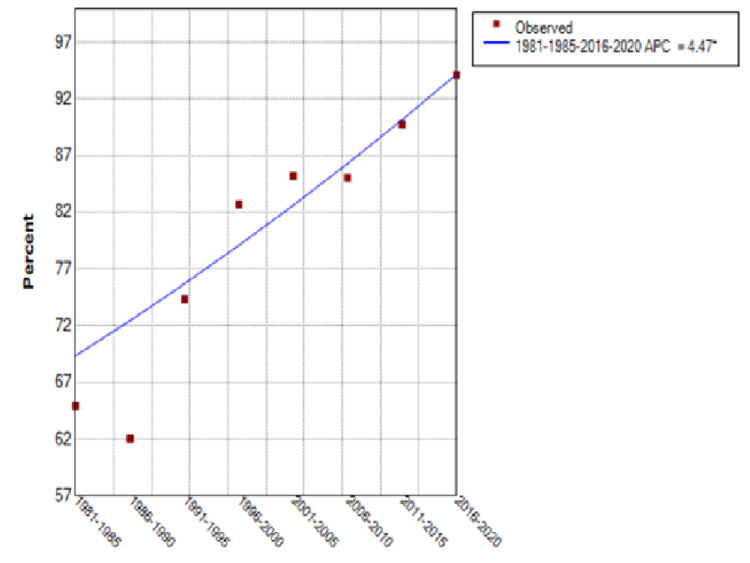
Patients With Known Follow-Up FAPTP: Florida Association of Pediatric Tumor Programs; APC: Annual percentage change

**Figure 7:**
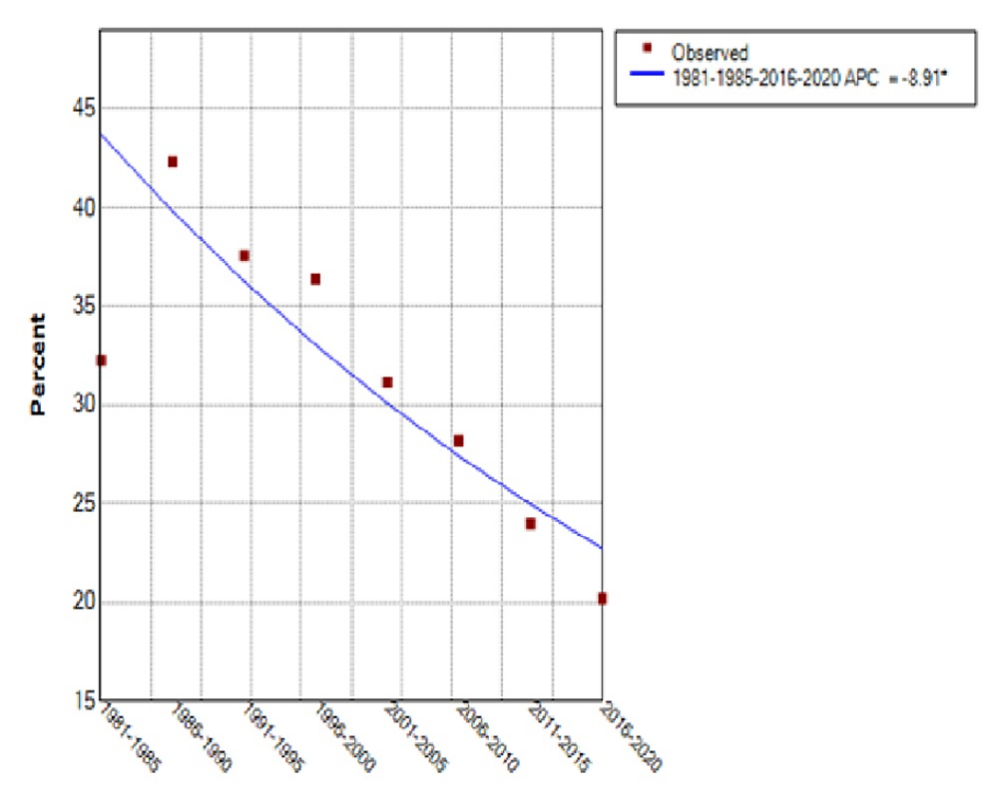
Patients Enrolled on Cooperative Group Studies FAPTP: Florida Association of Pediatric Tumor Programs; APC: Annual percentage change

From 1981 through 2020, the percentage of patients treated at FAPTP centers increased from 30% to 57% with an annual percentage change (APC) of 10.3% (95% Confidence Interval [CI] of 0.6 to 20.9%) (see Figure 5). There was a significant upward trend in the curve until around 2001, after which a plateau was noted. Those patients with known follow-up after completion of treatment rose from 65% to 94%, an APC of 4.5% (95% CI of 3 to 6%) (see Figure 6). The total number of patients enrolled on large cooperative group trials (CCG/POG/COG) increased 144% over this time, but the rate of clinical trial enrollment for established patients decreased from 32% in 1981-1985 to 20% for the period ending in 2020, after a peak of 42% in 1986-1990, for an APC of -8.91% (95% CI of -13.3 to -4.3%) (see Figure 7).

## Discussion

These data demonstrate a striking increase in reported pediatric cancer cases in Florida over the last 40 years that was much greater than could be accounted for by population growth. When compared to national data, the same trend is found. Although not an ideal comparison as it is based on a slightly different age range and time frame, the Centers for Disease Control published a review of pediatric cancer incidence (birth to 20) for the years 2003-2014 by region and state and found Florida’s incidence of new cases to be 17.0 per 100,000 people over that entire time block [6], while we noted an increase from 13.63 new cases per 100,000 people in 1981-1985 to 23.71 new cases per 100,000 people in 2016-2020. Looking past 2014 in the SPIRS database shows the incidence has increased even further in recent years. The simplest explanation is that children in Florida are truly getting cancer at a higher rate, irrespective of other factors, but it is most likely multifactorial. One reason is that there is improved reporting state-wide with the increase in the number of FAPTP centers during this time. There were surely a few smaller clinics outside of FAPTP seeing pediatric oncology patients from the 1980s onwards, but we do not feel that this accounted for a significant patient number and therefore was not a factor in the substantial increase in the number of patients registered in SPIRS over time. In addition, more advanced imaging techniques lead to the discovery of more incidental benign tumors, which would be reportable and could skew the data. Previous analysis of this database in 2010 demonstrated cancer diagnosis clusters in certain southeast and northern parts of the state, which would make environmental causes more suspicious for this rising incidence [7].

When examining the changing demographics of the state, another potential contributing factor comes to light: the most commonly diagnosed childhood cancer is acute lymphoblastic leukemia, and this disease is roughly 30% more common in Hispanics than in non-Hispanic whites and nearly 2-fold more common in Hispanics than in African Americans [8-10]. These differences were noted in the populations of both Florida in 2000 [8] and California in 2016 [9] and their respective incidences are nearly identical. Compounding this with the increasing number and proportion of Floridians that are Hispanic since 1980, we believe this factor cannot be understated. We also do acknowledge at the same time that our data is affected by database racial and ethnic classifications that are less specific than we would like. Florida, much like the rest of the country, has become very much more ethnically diverse over the last 40 years and within each rigid category such as “black” or “Hispanic,” there is much overlap, creating difficulty in an accurate depiction of the true ethnic and racial breakdown across the country and in Florida. We were not able to estimate APCs by race/ethnicity since we don’t know the proportion of each race that was treated at FAPTP centers, with known follow-up or on a COG study. The data used for analyses were aggregate data for all patients combined.

Yet another potential reason contributing to the increase in the number of oncology patients registered is that starting in approximately 2007, proton beam centers in Jacksonville and later Orlando began to receive referrals from outside the state, including international patients. In just the Jacksonville site alone, from 80-187 referrals were seen per year prior to a slow-down from the COVID-19 pandemic (Dr. Scott Bradfield, personal communication, Sept. 8, 2021). One final factor that could be contributing to the increase in patients is increased referrals of new or relapsed children from Latin America and the Caribbean islands, many of whom come to Florida for treatment because of its proximity.

The significant increase in raw numbers and the percentage of these patients receiving care at FAPTP centers should equate to more standardized care with specialist expertise as well as greater access to clinical trials. Although the overall number of patients enrolled on large cooperative group trials increased, after a peak in clinical trial enrollment to 42% of all known patients through 1990, we have noted a steady decline since, down to 20% in the most recent five-year block of data.

So, while there has been improvement in access to specialized care for these patients, this has not translated into a higher rate of clinical trial enrollment, which merits further investigation and ongoing initiatives. The SPIRS data only accounts for CCG/POG/COG clinical trials, so a possible contributor to this trend could be patients enrolling on clinical trials outside of these large cooperative groups. Examples could include intra-institutional and pharmaceutical-led trials either at a FAPTP center or a larger referral cancer center outside the state of Florida after initial diagnosis. In such a case, the child would be enrolled on a clinical trial, but that data would not be captured by the SPIRS database.

Although the SPIRS data is not powered to allow us to know many of the reasons for patients not being enrolled, many reviews of barriers to clinical trial enrollment have been published, and the most common cause is usually found to be the lack of open upfront therapeutic trials [11-13]. Some of that lack of availability may be because of the cooperative groups’ success in curing some of the most common diagnoses we see in pediatric oncology, such as standard risk acute lymphoblastic leukemia and Wilms tumor with what has become more standardized regimens over the years. Other researchers that have looked at COG clinical trial enrollment trends have similar hypotheses such as difficulty designing trials for malignancies with high cure rates and having enough trials to cover all solid and liquid diagnoses [14]. This was not the case in the earlier years of the study period, and these reduced enrollments may simply reflect the successes of earlier efforts of the cooperative groups to establish better regimens.

And even though the average age of SPIRS patients has gone up over the last 40 years, it has not risen enough within the notoriously under-enrolled AYA range to explain the lower clinical trial enrollment by itself. We were able to compare our data to COG-wide data over a similar time period, as Faulk et al. looked at the SEER data and contemporaneous COG enrollment on therapeutic trials for children aged 0 to 19 years from 2004-2015. They found a similar trend: by 2015, only 19.9% of cancer patients enrolled on a COG therapeutic trial [14], down from the 26.8% seen in another study looking at 2000 and 2003 [11]. In further analyzing these data from 2000 to 2003, Lund et al. found that the most underrepresented groups in COG trials were younger black and Hispanic children, Hispanic females, and white teenagers aged 15-19 [11]. Florida’s growing percentage of Hispanic children in the last 40 years may also be reflected in the enrollment data in this way. We cannot estimate APCs by race/ethnicity since we don’t know the proportion of each race that was treated at a FAPTP center with known follow-up or on a COG study. The data used for analyses were aggregate data for all patients combined. In addition, health insurance status and the policies of specific carriers also may impact on clinical trial enrollment, as in recent years, some insurance carriers have been less enthusiastic covering patients enrolled on clinical trials, deeming it “experimental.”

Unfortunately, the SPIRS data does have other limitations in addition to not measuring clinical trial enrollment outside of the large cooperative groups. One is that the clinical trial enrollment data does not separate biological from therapeutic studies. The rates of enrollment between ages and racial/ethnic groups are also lacking, making it impossible to compare directly with Lund’s findings. There are undoubtedly disparities in access to healthcare in the United States which would include access to and enrollment on clinical trials, but unfortunately, the SPIRS database does not have the appropriate data set to shed light on these disparities. Another weakness is that outcomes are not tracked, so we do not know if greater access to specialized cancer care at FAPTP centers has led to improved overall and event-free survival in this patient population. There has been overall improved survival among pediatric cancer patients nationally over this time period [15], so it is very likely that the children of Florida have benefited along with other children across the country, but we do not have this specific outcome in our data set. It would also be interesting to have the racial breakdown of the patients seen at FAPTP centers in the database, to see if access to these centers was equal among black, white and Hispanic children in Florida. In this vein, St. Jude’s Children Research Hospital published their data from 2001 to 2007 and compared it to SEER data over the same time. They found that there were disparities in outcomes with some pediatric cancers such as neuroblastoma and acute myeloid leukemia between black and white children on the national level, but not in their own cohort, where all children were treated on the same regimens and insurance, or lack thereof did not impact care [16]. To have such data on a statewide level would add to the depth of our findings.

## Conclusions

As the population of Florida has grown since 1980, so has the number of reported pediatric cancer patients in the state disproportionately along with the number of FAPTP centers. Our review of the unique SPIRS database, despite its limitations, shows that even though more children with cancer are being seen in these centers, there is still work to be done. Further mining of the data may allow us to better understand this population but improving access to top-quality cancer care across the state and addressing the racial, ethnic and age disparities, as well as dropping clinical trial enrollments would almost certainly lead to better outcomes, all of which should be measured prospectively moving forward.
